# Dynamics of Leukocyte Telomere Length in Patients with Fabry Disease

**DOI:** 10.3390/biomedicines12081724

**Published:** 2024-08-01

**Authors:** Tina Levstek, Nika Breznik, Bojan Vujkovac, Albina Nowak, Katarina Trebušak Podkrajšek

**Affiliations:** 1 Institute of Biochemistry and Molecular Genetics, Faculty of Medicine, University of Ljubljana, 1000 Ljubljana, Slovenia; 2 Clinical Institute for Special Laboratory Diagnostics, University Children’s Hospital, University Medical Centre Ljubljana, 1000 Ljubljana, Slovenia; 3 Centre for Fabry Disease, General Hospital Slovenj Gradec, 2380 Slovenj Gradec, Slovenia; 4 Department of Endocrinology and Clinical Nutrition, University Hospital Zurich, University of Zurich, 8091 Zurich, Switzerland; 5 Department of Internal Medicine, Psychiatry University Clinic Zurich, 8091 Zurich, Switzerland

**Keywords:** Fabry disease, telomere length, hypertrophic cardiomyopathy, nephropathy, stroke

## Abstract

Fabry disease (FD) leads to significant morbidity and mortality, which may indicate accelerated ageing. However, it is still unclear whether there is a relationship between telomere length (TL), a marker of biological ageing, and disease outcome. We aimed to examine the relationship between leukocyte TL (LTL) dynamics and the presence of advanced disease stages and/or late complications of FD, including hypertrophic cardiomyopathy, nephropathy and stroke, both cross-sectionally and longitudinally. DNA was extracted from peripheral blood leukocytes and quantitative PCR was utilized to determine relative LTL in 99 Fabry patients. In the longitudinal analysis, we included 50 patients in whom at least three measurements were performed over a period of 5–10 years. The results showed a significant inverse correlation between LTL and age (ρ = −0.20, *p* = 0.05). No significant differences in LTL were found between females and males (*p* = 0.79) or between patients receiving disease-specific therapy and those without (*p* = 0.34). In a cross-sectional analysis, no association was found between the presence (*p* = 0.15) or number (*p* = 0.28) of advanced stages of the disease and/or late complications and LTL. Similarly, in a longitudinal analysis, no difference in LTL dynamics was found regarding the presence (*p* = 0.16) of advanced stage organ involvement and/or late complications or their number. These findings indicate that LTL dynamics in adulthood may not be a reliable indicator of disease outcomes in Fabry patients. Therefore, LTL may more accurately reflect the disease burden in early life, when TL is primarily determined.

## 1. Introduction

Fabry disease (FD) is a rare genetic lysosomal disorder characterized by an accumulation of glycosphingolipids due to the insufficient activity of α-galactosidase A [1]. The accumulation of globotriaosylceramide (Gb3) in various cells and the extracellular accumulation of globotriaosylsphingosine (lyso-Gb3), a soluble form of Gb3, leads to tissue damage [2,3,4]. Clinical manifestations in females range from asymptomatic to severe, due to X-linked inheritance and lyonization [5,6,7]. Established disease-specific therapies (DSTs) for FD include replacing the deficiency of endogenous α-galactosidase A with a recombinant enzyme or increasing the activity of the enzyme with chaperones [8]. Despite the availability of these treatments, Fabry patients still have a shorter life expectancy than the general population, with untreated males living up to 20 years less and females up to 5 years less [9]. FD is therefore thought to be associated with premature ageing [10].

Telomere length (TL) is considered one of the main hallmarks of ageing, as it decreases with age [11]. In humans, telomeres consist of non-coding tandem TTAGGG DNA sequences that cap the end of chromosomes [12]. Their main function is to maintain the integrity of the genome by preventing DNA damage recognition at the end of chromosomes and the loss of genetic information [13]. However, telomeres shorten with each cell division due to the end replication problem [14]. The shortening continues until they reach a critically short length. Then, replicative senescence occurs, which leads to a permanent cell cycle arrest [15]. Telomere shortening is associated with various age-related diseases [16] and may increase the risk of overall and cause-specific mortality [17,18]. The role of telomere shortening in kidney diseases has also been investigated, but the results are inconclusive [19].

FD is associated with increased inflammation and oxidative stress, which may have a negative impact on TL [20]. Therefore, a link between the presence of a severe phenotype and faster telomere shortening may be biologically plausible. However, studies on TL in patients with FD are scarce. A recent study found shorter TL in men compared to age-matched controls [21]. However, there are currently no longitudinal studies that have measured TL over time and investigated the association between changes in TL and the occurrence of advanced disease stage and/or complications, particularly hypertrophic cardiomyopathy (HCMP), kidney dysfunction and stroke in patients with FD. The aim of the study is to investigate the relationship between leukocyte TL (LTL) and the occurrence of advanced disease stage and/or complications in a cohort of Fabry patients by performing both cross-sectional and longitudinal analyses over a period of up to 10 years. Understanding the role of TL shortening may help to elucidate the role of premature ageing in Fabry patients, believed to reduce their life expectancy and contribute to the disease progression [21].

## 2. Materials and Methods

### 2.1. Study Participants

Fabry patients were recruited from the Centre for Fabry disease of the General Hospital Slovenj Gradec in Slovenia and the Department of Endocrinology and Clinical Nutrition of the University Hospital Zurich in Switzerland. The group comprised of 33 Slovenian patients and 66 Swiss patients, all of Caucasian origin. The inclusion criteria were an age over 18 years, genetically confirmed FD, and at least three follow-ups in the last five years. Exclusion criteria for this study included uncontrolled hypertension, additional glomerular disease, kidney replacement therapy, a physical or mental illness that could interfere with the normal conduct of the study, pregnancy at the time of enrollment or within the last 12 months, and current or recent alcohol or drug abuse. We also included chronologic samples from 31 Slovenian and 19 Swiss patients collected during follow-up visits over the past decade. A total of 200 samples were included.

The patients were followed up regularly and underwent routine laboratory and clinical examinations. The presence of HCMP was assessed using echocardiography and/or cardiac MRI [22]. Progressive nephropathy was defined as an eGFR slope decline > 3 mL/min/1.73 m^2^/year [23]. Kidney function was estimated using the Chronic Kidney Disease Epidemiology Collaboration (CKD-EPI) equation [24]. Stroke was confirmed through appropriate imaging assessment.

The study protocol was approved by the Slovenian Ethics Committee for Research in Medicine (0120-260/2020/6, 0120-521/2020/3, and 0120-260/2020/12). All participants provided written informed consent in accordance with the Declaration of Helsinki.

### 2.2. Sample Collection and Isolation of Genomic DNA

Venous blood samples were collected in ethylenediaminetetraacetic acid (EDTA) tubes and stored at −80 °C. Chronological blood samples were taken at intervals of approximately 2–3 years during routine examinations over the last 10 years and stored at −80 °C. Only patients from whom at least three samples were available were considered.

The genomic DNA was extracted from EDTA samples using the FlexiGene DNA Kit 250 (Qiagen, Hilden, Germany) following the manufacturer’s instructions. The quantity and quality of the DNA samples were determined spectrophotometrically using the NanoDrop One (Thermo Fisher Scientific, Waltham, MA, USA). The isolated DNA was stored at 4 °C.

### 2.3. Measurement of Telomere Length by Quantitative PCR

Before further analysis, the DNA was diluted to a concentration of 15 ng/µL. Multiplex quantitative PCR (qPCR) was performed to determine TL as previously described [25,26]. An 8.7 µL master mix was prepared using 1× Melt Doctor HRM MasterMix (Thermo Fisher Scientific, Waltham, MA, USA), the primers TelG (ACACT AAGGT TTGGG TTTGG GTTTG GGTTT GGGTT AGTGT), TelC (TGTTA GGTAT CCCTA TCCCT ATCCC TATCC CTATC CCTAACA), AlbF (CGGCG GCGGG CGGCG CGGGC TGGGC GGAAA TGCTG CACAG AATCC TTG) and AlbR (GCCCG GCCCG CCGCG CCCGT CCCGC CGGAA AAGCA TGGTC GCCTGTT) (Integrated DNA Technologies, Coralville, IA, USA), and RNase/DNase-free water. To the master mix, 1.3 µL of diluted DNA was added to reach a final volume of 10 µL. Each reaction contained 20 ng of DNA and 0.3 µM of each primer. TL measurements were performed using the QuantStudio 7 Flex Real-Time PCR System (Applied Biosystems, Waltham, MA, USA). The cycle design was as follows: 95 °C for 15 min; 94 °C for 15 s, 49 °C for 15 s, for 2 cycles; 94 °C for 15 s, 62 °C for 10 s, 74 °C for 15 s, 84 °C for 10 s, and 88 °C for 15 s, for 32 cycles. The melting curve was established by ramping from 60 to 95 °C with an increase of 0.05 °C/s. Samples were measured in triplicate, together with a no template control and a positive control, both measured in quadruplicate. Using Human Genomic DNA (Roche, Basel, Switzerland) at a dilution of 1:3, a five-point standard curve was generated, ranging from 150 to 1.85 ng/µL. After thermal cycling and raw data acquisition, two standard curves were generated using QuantStudio™Real-Time PCR Software v1.3, one for telomere signal (T) and one for albumin signal (S). Only samples with an SD of less than 0.1 for T and S in the replicates were used to calculate the T/S ratio.

### 2.4. Statistical Analysis

All analyses were performed using RStudio version 4.3.0 (R Core Team, Vienna, Austria). For the descriptive statistical analysis, the normality of the distribution of the continuous variables was tested using the Shapiro–Wilk test. The median with interquartile range (25–75%) or the mean with standard deviation were used to describe the central tendency and variability of the continuous variables. The frequencies were used to describe the distribution of categorical variables. Two-tailed *p* values < 0.05 were considered significant. A total of 99 patients were included, exceeding the calculated sample size of 83. The sample size calculation was performed using the pwr package, assuming a small effect size (d = 0.1), a test power of 80% and an alpha level of 0.05.

Correlation was tested using the Pearson correlation test. The two-sample Student’s *t*-test was used to compare TL for covariates with two categories, while a one-way ANOVA was used to assess covariates with three categories. ANCOVA was used for age-adjusted comparisons between groups.

Linear mixed-effects models were used to test the association between TL dynamics and the presence of advanced disease stage and/or complications over a 10-year period (lme4 package). All models included a random intercept for each patient to account for individual variability within the longitudinal data. The T/S values were logarithmically transformed. The models were visually validated using diagnostic plots, and model parameters were specified using models fitted with restricted maximum likelihood (REML). We only included predictor variables with an intercorrelation of Pearson’s r < 0.5 for all relevant pairs of explanatory variables.

## 3. Results

### 3.1. Patient’s Characteristics

The characteristics of the patients are listed in Table 1. In the cross-sectional cohort, 60 patients had stage 1, 28 had stage 2, 6 had stage 3, and 5 had stage 4 or 5 chronic kidney disease. Of the total number of patients, 69 patients had a urinary albumin-to-creatinine ratio (UACR) of less than 30 mg/g, 20 had a UACR between 30 and 300 mg/g, and 2 had a UACR greater than 300 mg/g (data for 8 patients were missing). Overall, 56 (56.6%) patients had neither advanced disease stage nor any complications, while 43 (43.4%) had an advanced disease stage and/or at least one complication or advanced stage of the disease. Only 2 patients (2.0%) had progressive nephropathy, HCMP, and stroke. Patients *GLA* genotypes are listed in Table S1.

A longitudinal analysis was performed on 50 Fabry patients who were followed up for a period of 5 to 10 years; their characteristics are listed in Table 1. Among those, only 2 (4%) patients had progressive nephropathy, HCMP, and stroke. None of the patients died during the follow-up period.

### 3.2. Cross-Sectional Analysis of Telomere Length in Fabry Patients

**Figure 1:**
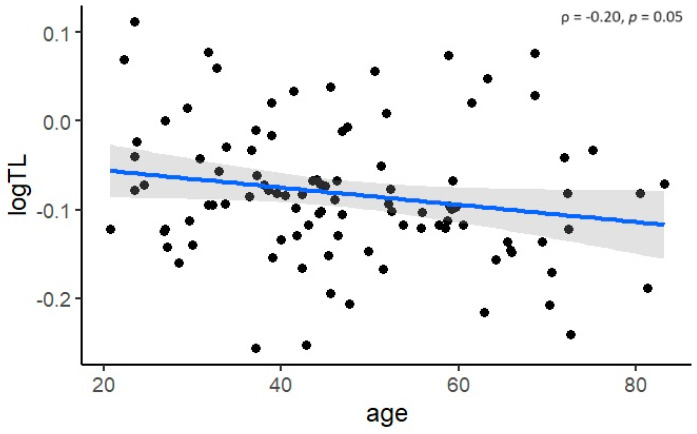
Negative correlation between telomere length (TL) and age.

**Figure 2:**
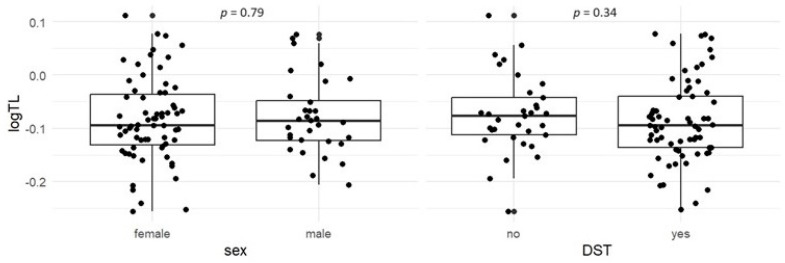
Comparison of telomere length (TL) between females and males and between patients receiving disease-specific therapy (DST) and those not receiving DST.

In the cross-sectional analysis, LTL was negatively associated with age (ρ = −0.20, *p* = 0.05) (Figure 1). However, no statistically significant difference in TL was observed between males and females (*p* = 0.79) or between patients with and without DST (*p* = 0.34) (Figure 2). Even after adjusting for age, the differences remained statistically insignificant (*p* = 0.81 and *p* = 0.74, respectively).

An ANCOVA was performed to assess the effect of the number of the advanced disease stage and/or complications on LTL, while controlling for age. After adjusting for age, there was no statistically significant difference in LTL between groups (F(2, 95) = 1.26, *p* = 0.28) when comparing patients with no advanced disease stage and/or complications, patients with one complication or advanced disease stage, and patients with two or three complications or advanced disease stages. Even after controlling for age, there was no statistically significant difference in LTL between patients with and without an advanced disease stage and/or complications (F(1, 96) = 2.07, *p* = 0.15).

### 3.3. Longitudinal Analysis of Telomere Length Dynamics in Fabry Patients

In the longitudinal analysis, we examined the impact of the number or presence of late complications or advanced disease stage on LTL dynamics. Both models demonstrated that LTL dynamics were significantly influenced by baseline LTL (*p* < 0.01). However, we did not observe a significant difference in TL dynamics between patients without an advanced disease stage and/or complications and those with one or more than two complications or advanced disease stages. Similarly, no significant difference was found between patients with an advanced disease stage and/or complications and those without (Table 2).

## 4. Discussion

Predicting the clinical course of FD is challenging due to the great variability of the clinical presentation. Therefore, better prognostic information is needed to guide the management of Fabry patients. The role of telomere biology in disease progression in Fabry patients and its potential use as a biomarker for the disease remains elusive. This study is the first to investigate the association between LTL dynamics and the occurrence or number of advanced disease stages and/or late complications in patients with FD, both cross-sectionally and longitudinally. The cross-sectional analysis revealed no associations between LTL and advanced disease stage and/or complications. The longitudinal analysis yielded similar results, suggesting that there is no relationship between the development of an advanced disease stage and/or complications and LTL dynamics.

Previous studies in the general population have shown that TL shortens by 50–150 bp per year [14]. Therefore, a significant negative correlation between LTL and age, as found in our study, was to be expected. Other factors that have been reported to influence LTL include sex, with studies showing that LTL is equivalent between boys and girls at birth [27], but longer in females than in males in adulthood. However, sex-related differences in LTL have been found to be more difficult to detect in younger adults [28]. Furthermore, a meta-analysis concluded that only studies using the Southern blot method showed that females have longer telomeres than males [29]. A recent study showed that LTL were significantly shorter in patients with FD compared to matched healthy controls, mainly due to LTL difference in males [21]. In our group of patients, with a mean age of 47.3 years, no difference in LTL was found between males and females. We found no difference in LTL between patients receiving DST and those not receiving therapy, which is consistent with a previous study showing that enzyme replacement therapy (ERT) has no effect on telomere attrition [21]. This may be partly due to the fact that ERT may mitigate disease progression but cannot completely prevent organ damage progression in Fabry patients [30,31]. On the other hand, patients who receive DST are typically more severely affected than those who receive no therapy. Therefore, the lack of correlation could also indicate that DST stabilizes LTL. However, further studies are necessary to investigate the effect of DST on LTL dynamics.

The association between a short LTL and various conditions has been extensively studied in recent years. However, the results are often contradictory and mostly based on cross-sectional studies [32]. In our cohort of Fabry patients, we found no significant difference in LTL in relation to the presence of an advanced stage of organ involvement and/or the presence and number of late complications. However, cross-sectional studies alone cannot establish a causal relationship between LTL and clinical outcomes related to ageing, as associations may have originated in childhood or be confounded by non-age-related differences between older and younger cohorts. Therefore, we also conducted a longitudinal analysis in which LTL was measured at least three times over a period of up to 10 years. Our longitudinal study found no statistically significant association between LTL shortening and the presence of an advanced stage of organ involvement and/or presence and number of late complications in Fabry patients. This finding confirms the conclusion of some other previous studies that TL might not be a useful biomarker for age-related risks such as multimorbidity, mortality, cognitive decline, etc. [32,33,34,35]. In agreement with previous studies, we also found that changes in LTL dynamics during follow-up were impacted by baseline LTL [17,36,37].

It is noteworthy that the rate of LTL shortening is considerably faster during infancy and early childhood than in adulthood. Furthermore, LTL is a heritable trait with a parental effect [38]. Studies on twins indicate that TL has a heritability of 36–78% [39,40]. In general, TL seems to be mainly determined before adulthood, with heritability and early life factors being the most important determinants of LTL throughout the lifespan [41]. A study of four adult cohorts found that the inter-individual ranking and tracking of TL remained stable over six decades, supporting the idea that the most of telomere variability arises in the early years of life [42]. Therefore, it is important to conduct further studies in infants and children to understand the role of telomere shortening in age-related diseases. This is particularly important in Fabry patients, as the accumulation of toxic products has already been observed in fetuses [43]. In individuals with the classic phenotype, the first symptoms usually manifest in early childhood. These symptoms include acroparesthesias, angiokeratomas, cornea verticillata, an/hypohidrosis, gastrointestinal problems, and heat intolerance [4,20]. Therefore, LTL may serve as a biomarker of how the disease burden in early life has affected the outcome in adulthood of Fabry patients. While premature ageing in FD might not be directly related to the accelerated TL attrition, other pathophysiological pathways of ageing need to be explored, such as methylation-based epigenetic clocks [44] or ribosomal DNA-based clocks [45].

Our study also has some limitations that should be considered when interpreting the results. We measured LTL, which is only a surrogate marker for TL in different tissues. It would be interesting to study TL in the affected organs, but obtaining tissue is invasive, unlike leukocytes, which are readily available. As FD is a rare disease, the number of patients that can be included in the study is quite limited. Our study design also did not allow for assessing the relationship between LTL and mortality risk in Fabry patients, which may be worth investigating in the future. The study also has several strengths, including its prospective design, the first of its kind, and the thorough phenotypic characterization of the patients, who were followed for 5–10 years. Furthermore, the characteristics of the patients included in the cross-sectional and longitudinal design were quite similar, as shown in Table 1. All samples were measured in triplicate, and only those with a standard deviation of less than 0.10 were included to minimize measurement error. DNA was isolated using the same protocol and at the same time. This is a crucial point, as the storage and isolation protocol can influence LTL [46].

## 5. Conclusions

Our study shows that telomere shortening has no significant impact on the occurrence of advanced disease stage and/or complications in adult patients with FD. These findings suggest that telomere shortening in adulthood does not have a major impact on disease progression, which is consistent with previous studies emphasizing the importance of determining TL in early childhood. Indirect associations between LTL and disease burden may be more plausible. In addition, DST showed no association with LTL dynamics.

## Figures and Tables

**Table 1:** Patient’s characteristics.

Parameter	Cross-Sectional Analysis (n = 99)	Longitudinal Analysis (n = 50)
Males (%)	32 (32.2)	16 (32.0)
Age (years)	47.3 ± 15.3	43.1 ± 15.1
DST	66 (66.7)	29 (58.0)
HCMP	37 (37.4)	20 (40.0)
Progressive nephropathy	15 (15.2)	8 (16.0)
Stroke	8 (8.1)	4 (8.0)

DST, disease-specific therapy; HCMP, hypertrophic cardiomyopathy.

**Table 2:** Linear mixed models for the assessment of LTL dynamics depending on the number or presence of late complications or advanced stages of disease.

	Estimate	St. Error	*p* Value
Model 1			
Years	−0.0030	0.0016	0.07
Baseline LTL	0.2006	0.0539	<0.01
Age	−0.0004	0.0005	0.40
Group 1 vs. group 2	−0.0266	0.0154	0.09
Group 1 vs. group 3	−0.0058	0.0196	0.77
Model 2			
Years	−0.0030	0.0016	0.05
Baseline LTL	0.2060	0.0535	<0.01
Age	−0.0003	0.0005	0.49
Group 1 vs. group 4	−0.0199	0.0139	0.16

In model 1, patients without advanced disease stage and/or complications (group 1) were compared with patients with advanced disease stage and/or one complication (group 2) or patients with advanced disease stage and/or two or three complications (group 3). In model 2, patients without advanced disease stage or complications (group 1) were compared with patients with at least one complication or advanced stage of disease (group 4). LTL, leukocyte telomere length.

## Data Availability

Data are contained within the article and Supplementary Materials.

## Supplementary Materials

The following supporting information can be downloaded at: https://www.mdpi.com/article/10.3390/biomedicines12081724/s1, Table S1: Variants in the *GLA* gene of the included Fabry patients (reference sequence NM_000169.2).
