# Supplementary material for: Dynamics of Leukocyte Telomere Length in Patients with Fabry Disease
Source: Biomedicines. 2024 Aug 1;12(8):0. doi: 10.3390/biomedicines12081724 (PMC11351930; doi:10.3390/biomedicines12081724)
Supplement: Supplementary file 1 [file biomedicines-12-01724-s001.zip › Levstek_Supplementary Material.pdf]

Supplementary Material

Table S1. Variants in the *GLA* gene of the included Fabry patients (reference sequence NM_000169.2).

ID	Genetic variant		Population
1	p.Arg363Pro	c.1088G>C	Slovene
2	p.Arg363Pro	c.1088G>C	Slovene
3	p.Asn272Ser	c.815A>G	Slovene
4	p.Arg227Ter	c.679C>T	Slovene
5	p.Asn272Ser	c.815A>G	Slovene
6	p.Asn272Ser	c.815A>G	Slovene
7	p.Ile270Met	c.810T>G	Slovene
8	p.Asn272Ser	c.815A>G	Slovene
9	p.Leu180Phe	c.540G>C	Slovene
10	p.358delGlu	c.1072_1074delGAG	Slovene
11	p.Arg227Ter	c.679C>T	Slovene
12	p.Asn272Ser	c.815A>G	Slovene
13	p.Ile270Met	c.810T>G	Slovene
14	p.Asn272Ser	c.815A>G	Slovene
15	p.Asn272Ser	c.815A>G	Slovene
16	p.Asn272Ser	c.815A>G	Slovene
17	p.Arg342Gln	c.1025G>A	Slovene
18	p.Glu87Asp	c.261_278del18	Slovene
19	p.Asn272Ser	c.815A>G	Slovene
20	p.Asn272Ser	c.815A>G	Slovene
21	p.Arg363Pro	c.1088G>C	Slovene
22	p.Glu87Asp	c.261_278del18	Slovene
23	p.Arg49Pro	c.146G>C	Slovene
24	p.Arg49Pro	c.146G>C	Slovene
25	p.Arg342Gln	c.1025G>A	Slovene
26	p.Arg363Pro	c.1088G>C	Slovene
27	p.Arg363Pro	c.1088G>C	Slovene
28	p.Asn272Ser	c.815A>G	Slovene
29	p.Arg227Ter	c.679C>T	Slovene
30	p.Cys63Tyr	c.188G>C	Slovene
31	p.Asn272Ser	c.815A>G	Slovene
32	p.Asn272Ser	c.815A>G	Slovene
33	p.Arg227Ter	c.679C>T	Slovene
34	p.Cys172Arg	c.514T>C	Swiss
35	p.Arg227Gln	c.680G>A	Swiss
36	p.Asp266Tyr	c.796G>T	Swiss
37	p.Met42Thr	c.125T>C	Swiss
38	p.Arg301Gln	c.902G>A	Swiss
39	p.ins353Thr	c.1055_1057dupCTA	Swiss
40	p.Ser345Pro	c.1033T>C	Swiss
41	p.Asp266Tyr	c.796G>T	Swiss
42	p.Tyr194Ile	c.581C>T	Swiss

43	Asp266Tyr	c.796G>T	Swiss
44	p.Arg118Cys	c.352C>T	Swiss
45	p.Met290IIe	c.870G>C	Swiss
46	p.Val390Cysfs*9	c.1168insT	Swiss
47	p.ins353Thr	c.1055_1057dupCTA	Swiss
48	/	c.640-2A>G	Swiss
49	p.Trp24Ter	c.72G>A	Swiss
50	p.Thr194Ile	c.581C>T	Swiss
51	p.Met42Thr	c.125T>C	Swiss
52	p.His46Tyr	c.136C>T	Swiss
53	p.IIe317Thr	c.950T>C	Swiss
54	p.Asn215Ser	c.644A>G	Swiss
55	p.Thr194Ile	c.581C>T	Swiss
56	p.Met42Thr	c.125T>C	Swiss
57	p.delPhe383	c.1146delCTT	Swiss
58	p.Ser345Pro	c.1033T>C	Swiss
59	p.Thr194Ile	c.581C>T	Swiss
60	p.Val390CysfsTer9	c.1168insT	Swiss
61	p.Phe113Leu	c.337T>C	Swiss
62	exon 2 deletion	g.2962_5871del	Swiss
63	p.Thr194Ile	c.581C>T	Swiss
64	p.Phe248LeufsTer7	c.744_745delTA	Swiss
65	p.Phe113Leu	c.337T>C	Swiss
66	p.Arg301Gln	c.902G>A	Swiss
67	p.Met42Thr	c.125T>C	Swiss
68	p.Ser238Asn	c.713G>A	Swiss
69	p.Ser345Pro	c.1033T>C	Swiss
70	p.Met187ValfsTer6	c.559_560delAT	Swiss
71	p.Ser276Asn	c.827G>A	Swiss
72	p.Thr194Ile	c.581C>T	Swiss
73	p.Ter254	c.744_745delTA	Swiss
74	p.Ter254	c.744_745 delTA	Swiss
75	p.Arg301Gln	c.902G>A	Swiss
76	p.Val390CysfsTer9	c.1167dupT	Swiss
77	p.Arg301Ter	c.901C>T	Swiss
78	p.Ser345Pro	c.1033T>C	Swiss
79	p.Ser235Tyr	c.704C>A	Swiss
80	p.Phe113Leu	c.337T>C	Swiss
81	p.Val390CysfsTer9	c.1167dupT	Swiss
82	p.Phe113Leu	c.337T>C	Swiss
83	Ter254	c.744_745delTA	Swiss
84	p.Val390CysfsTer9	c.1167dupT	Swiss
85	p.Thr194Ile	c.581C>T	Swiss
86	p.Ser345Pro	c.1033T>C	Swiss
87	p.delPhe383	c.1146delCTT	Swiss
88	p.Met42Thr	c.125T>C	Swiss

89	p.Asn122IlefsTer8	c.364delA	Swiss
90	p.Phe113Leu	c.337T>C	Swiss
91	p.Phe113Leu	c.337T>C	Swiss
92	p.Thr194Ile	c.581C>T	Swiss
93	p.Thr194Ile	c.581C>T	Swiss
94	p.Phe113Leu	c.337T>C	Swiss
95	p.Ter254	c.744_745delTA	Swiss
96	p.Arg301Ter	c.901C>T	Swiss
97	p.Arg301Gln	c.902G>A	Swiss
98	p.Thr194Ile	c.581C>T	Swiss
99	p.Thr194Ile	c.581C>T	Swiss
